# Photoacoustic Tomography Detects Response and Resistance to Bevacizumab in Breast Cancer Mouse Models

**DOI:** 10.1158/0008-5472.CAN-21-0626

**Published:** 2022-04-11

**Authors:** Isabel Quiros-Gonzalez, Michal R. Tomaszewski, Monika A. Golinska, Emma Brown, Laura Ansel-Bollepalli, Lina Hacker, Dominique-Laurent Couturier, Rosa M. Sainz, Sarah E. Bohndiek

**Affiliations:** 1 Department of Physics, University of Cambridge, Cambridge, United Kingdom.; 2 Cancer Research UK Cambridge Institute, University of Cambridge, Li Ka Shing Centre, Cambridge, United Kingdom.; 3 Cell Morphology and Biology Department, IUOPA and ISPA, Universidad de Oviedo, Oviedo, Spain.

## Abstract

Angiogenesis is an established prognostic factor in advanced breast cancer, yet response to antiangiogenic therapies in this disease remains highly variable. Noninvasive imaging biomarkers could help identify patients that will benefit from antiangiogenic therapy and provide an ideal tool for longitudinal monitoring, enabling dosing regimens to be altered with real-time feedback. Photoacoustic tomography (PAT) is an emerging imaging modality that provides a direct readout of tumor hemoglobin concentration and oxygenation. We hypothesized that PAT could be used in the longitudinal setting to provide an early indication of response or resistance to antiangiogenic therapy. To test this hypothesis, PAT was performed over time in estrogen receptor–positive and estrogen receptor–negative breast cancer xenograft mouse models undergoing treatment with the antiangiogenic bevacizumab as a single agent. The cohort of treated tumors, which were mostly resistant to the treatment, contained a subset that demonstrated a clear survival benefit. At endpoint, the PAT data from the responding subset showed significantly lower oxygenation and higher hemoglobin content compared with both resistant and control tumors. Longitudinal analysis revealed that tumor oxygenation diverged significantly in the responding subset, identifying early treatment response and the evolution of different vascular phenotypes between the subsets. Responding tumors were characterized by a more angiogenic phenotype when analyzed with IHC, displaying higher vessel density, yet poorer vascular maturity and elevated hypoxia. Taken together, our findings indicate that PAT shows promise in providing an early indication of response or resistance to antiangiogenic therapy.

**Significance:**

Photoacoustic assessment of tumor oxygenation is a noninvasive early indicator of response to bevacizumab therapy, clearly distinguishing between control, responding, and resistant tumors within just a few weeks of treatment.

## Abstract (precis)

Photoacoustic assessment of tumor oxygenation is a noninvasive early indicator of response to bevacizumab therapy, clearly distinguishing between control, responding, and resistant tumors within just a few weeks of treatment.

## Introduction

The dynamic cellular ecosystem of a growing tumor mass requires a vascular network (1) to obtain oxygen and nutrients, as well as to remove metabolic waste products (2). Early in tumor development, blood vessel growth is stimulated through angiogenesis, which is recognized as a cancer hallmark (3). The notion that angiogenesis enables progression of solid tumors led to the development and approval of numerous antiangiogenic therapies (4); however, their efficacy has been highly variable between cancer sites and disease subtypes, and is often transitory (5).

Angiogenesis is a prognostic factor in advanced breast cancer, hence early in their development, antiangiogenic therapies were trialed in the disease, alone and in combination with chemotherapy (6). Blockade of VEGF using the mAb bevacizumab received European Medicines Agency (EMA) and FDA approvals in 2007 and 2008, respectively, for use in patients with metastatic HER2-negative breast cancer after it was shown to nearly double progression-free survival in the E2100 trial (7, 8). Subsequent trials failed to demonstrate the conversion of initial progression-free survival benefit into an increase in overall survival, leading to withdrawal of the FDA approval in 2011 (9).

A key challenge in the application of antiangiogenic therapies in breast cancer is the lack of specific biomarkers for identifying those patients in which the therapy could be efficacious and guiding dosing regimens with chemotherapy (10) or metronomic therapy (11). Most prior clinical trials have been conducted in unselected populations, which fail to account for the substantial heterogeneity of breast cancer (12), yet prior work that did consider patient heterogeneity found that pretreatment microvessel density (MVD) could be a potential predictive biomarker of response in the neoadjuvant setting (13). Nonetheless, even within a single tumor, vessels can differ in their susceptibility to antiangiogenic therapy, leading to both intrinsic and acquired resistance (4, 14, 15).

Noninvasive imaging biomarkers derived from the patient tumor vasculature could help to address these challenges, by identifying those patients and tumors with expected susceptibility to antiangiogenic therapy and monitoring therapeutic response longitudinally, enabling dosing regimens to be altered with real-time feedback. MRI has already been widely applied for quantification of microvascular structure and function in phase I/II clinical trials of antiangiogenic therapies. In particular, dynamic contrast enhanced (DCE) MRI biomarkers, related to vascular perfusion and permeability, yield a dose-dependent response and correlate to progression-free survival (10). Nonetheless, a lack of multicentre standardization in acquisition and analysis for DCE-MRI methods, combined with the significant cost of implementing complex imaging procedures in larger patient cohorts (16), are obstacles for widespread use (10). State-of-the-art, localized imaging modalities that can provide similar insight at lower cost may thus have a role to play in predicting and monitoring antiangiogenic therapy response.

Photoacoustic tomography (PAT) is an emerging modality that has shown promise in clinical trials for breast cancer diagnosis (17). PAT exploits the absorption of light by hemoglobin, yielding intrinsic sensitivity to the concentration and oxygenation of hemoglobin in tissues, providing both structural and functional insight into the tumor vasculature (18). PAT has shown promise in detecting treatment response in cancer across several classes of therapy (19–21). In the context of antiangiogenic therapy, photoacoustics has already been shown in murine tumor models to be intrinsically sensitive to disruption of vessel architecture (22, 23) and improvements in oxygenation (24–26) associated with vessel normalizing effect of angiogenesis blockade. Furthermore, when combined with a contrast agent, PAT perfusion measurements during antiangiogenic treatment decrease in response to vascular pruning (27) and increase with elevated vascular permeability (28). Nonetheless, the ability of PAT to predict response to antiangiogenic therapy has yet to be investigated.

We hypothesized that the exquisite sensitivity of PAT to characterize tumor vascular evolution (29, 30) could be exploited to identify response and resistance to antiangiogenic therapy. Here, we performed longitudinal imaging of two human breast cancer xenograft models undergoing treatment with the antiangiogenic drug bevacizumab as a single agent and characterized the underlying biological changes using biochemical and IHC markers. We demonstrate that PAT assessment of oxygenation indicates survival benefit in a subset of these tumors and correlates with underlying changes in tumor vascular biology.

## Materials and Methods

### Cell lines

The human adenocarcinoma cell lines MCF7 (estrogen receptor positive) and MDA-MB-231 (estrogen receptor negative) were obtained from the Cancer Research UK (CRUK) Cambridge Institute Biorepository from the University of Cambridge (Cambridge, United Kingdom). Experiments were performed when cells were between passage 20–25 for both MCF7 and MDA-MB-231. *Mycoplasma* testing was performed annually. Authentication using Genemapper ID v3.2.1 (Genetica) by STR Genotyping (1/2015) showed 100% match with the reference sequence in both cases. Cells were maintained in DMEM supplemented by 10% of FBS at 37°C in 5% CO_2_.

### *In vivo* models and dosing regimen

**Figure 1.**
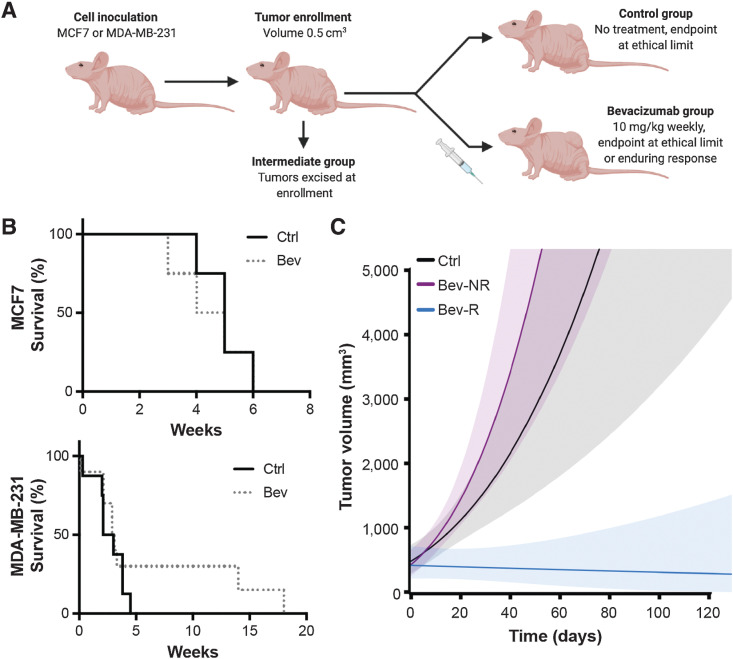
Bevacizumab provides a survival benefit in a subset of MDA-MB-231 tumors but not MCF7 tumors. **A,** Illustrative overview of study design (created with BioRender.com). Mouse viewpoint from left side shows only the left flank tumor; right flank tumor not shown. **B,** Survival curves for control (Ctrl) and bevacizumab (Bev)-treated groups in MCF7 tumor-bearing mice (top, *n*_mice_ = 4 Ctrl; *n*_mice_ = 4 Bev) and MDA-MB-231 tumor-bearing mice (bottom, *n*_mice_ = 8 Ctrl; *n*_mice_ = 11 Bev). **C,** Estimated average tumor growth per group for the control group and the bevacizumab-treated group of MDA-MB-231 mice (*n*_tumors_ = 14 Ctrl; *n*_tumors_ = 20 Bev), illustrating the durable response obtained in the subset of the treated group denoted as responders (Bev-R) compared with nonresponders (Bev-NR) based on their tumor growth rates (see Supplementary Data). Shaded areas show 95% pointwise confidence bounds for the average. The largest tumor volume recorded in the study was less than 3,200 mm^3^ in accordance with our ethical limits; data beyond this volume is an extrapolation of our linear model to illustrate the trajectory of growth in Ctrl and Bev-NR tumors. Weeks denoted in **B** and **C** are from the time of enrollment.

All animal procedures were conducted in accordance with project (70-8214) and personal license (IB8699B5B) issued under the United Kingdom Animals (Scientific Procedures) Act, 1986 and were approved locally under compliance form (CFSB0703). An overview of the study design is provided in Fig. 1A. Seven-week-old immunodeficient female nude (BALB/c nu/nu) mice (*n*_MDA-MB-231_ = 23; *n*_MCF7_ = 14; Charles River) were inoculated in the mammary fat pad in both flanks (10^5^ cells per flank of the same cell line per mouse) in a final volume of 100 µL of 1:1 DMEM (Gibco) and Matrigel (BD Biosciences), therefore each experimental group bears one type of breast cancer cell line. For MCF7, endogenous estrogen levels were supplemented by surgical implantation of an estrogen pellet (0.72 mg/pellet, 90 days release; Innovative Research of America) in the scruff. Tumor growth was monitored weekly by callipers. When tumors reached a volume of 0.5 cm^3^, animals were assigned randomly to any of the following groups: intermediate, control (Ctrl) and bevacizumab treated (Bev). The intermediate group (number of mice *n*_MDA-MB-231_ = 4, *n*_MCF7_ = 6) was immediately euthanized, and the tissues and serum collected. The Bev group was then treated weekly with the antiangiogenic drug bevacizumab, administered once per week intraperitoneally at a clinically scaled dose of 10 mg/kg. The dose was selected on the basis of the clinical dosage recommendation of 5–15 mg/kg every 2 or 3 weeks in humans, with weekly dosing used here to account for the shorter lifespan and faster tumor development in the mouse. Endpoint was defined as when tumor size exceeded 1.5 cm diameter, or tumor mass was equivalent to 10% of the animal body weight. For tumors that achieved an enduring response to the therapy, endpoint was defined as 6 months after tumor inoculation. At endpoint, animals were euthanized and tumors and serum were collected.

### PAT

A commercial small animal PAT system was [multispectral optoacoustic tomography (MSOT) inVision 256-TF; iThera Medical GmbH]. The system has been described in detail elsewhere (31, 32). Briefly, a tunable (660–1,300 nm) optical parametric oscillator, pumped by a nanosecond (ns) pulsed Nd:YAG laser, with 10 Hz repetition rate and up to 7 ns pulse duration is used for signal excitation. Light is delivered to the sample through a custom optical fibre assembly to obtain a uniform diffuse ring of illumination over the imaging plane. Coupling of the sample to the transducers is achieved using a water bath, filled with degassed heavy water (D_2_O). An array of transducers covering an angle of 270° forms the detector, allowing tomographic reconstruction.

Mice were imaged weekly after enrollment and prepared according to our previously published standard operating procedure (33). Briefly, mice were anaesthetized using <3% isoflurane and placed in a custom animal holder (iThera Medical), wrapped in a thin polyethylene membrane, with ultrasound gel (Aquasonic Clear, Parker Labs) used to couple the skin to the membrane. The holder was then placed within the PAT system and immersed in degassed D_2_O maintained at 36°C. The animal respiratory rate was maintained in the range 70–80 bpm with approximately 1.5% isoflurane concentration for the entire scan. The animal holder was translated along the oral-caudal axis of the tumor and serial images every 0.5 mm were taken for all the animals. Images were acquired using wavelengths between 700 nm and 1,100 nm (700, 725, 750, 775, 800, 825, 850, 900, 920, 950, 970, 1,000, 1,040, and 1,100 nm), with an average of 10 pulses per wavelength. Each slice took 14 seconds to acquire.

### IHC

Tissues were collected and fixed in 4% paraformaldehyde for 24 hours. Samples were processed by the Cancer Research UK Cambridge Institute Histopathology Core. Tumor tissues were embedded in paraffin, sectioned consecutively at 3 µmol/L thickness through the central level of the tumor, and sections were then rehydrated. Hematoxylin and eosin (H&E) and toluidine blue stains were applied to two sections. IHC was performed using Polymer Refine Detection System (DS9800) in a BOND automated stainer (Leica Biosystems) for mouse CD31 (1:50; 553370, BD Biosciences), alpha smooth muscle actin (1:500; ASMA; ab5694, Abcam) and F4/80 (1:20, MCA497, Serotec), and human VEGF (1:200; RB-9031, Thermo Fisher Scientific) and carbonic anhydrase IX (1:250) CA-IX; AB1001, Biosciences Slovakia). All of the antibodies required antigen retrieval. Antigen retrieval was performed prior to primary antibody incubation as follows: 10′ Proteinase K pretreatment at 37°C for CD31, 10′ Tris-EDTA pretreatment at 100°C for ASMA, and 20′ sodium citrate pretreatment at 100°C for VEGF and CA-IX.

For hypoxyprobe staining, the pimonidazole probe (Hypoxyprobe, Inc.) was injected intraperitoneally at 60 mg/kg at 45 minutes before euthanasia of the mouse. Samples were collected, fixed, and processed as indicated above with antigen retrieval requiring 20′ Tris-EDTA treatment.

### Blood and serum measurements

For biochemical analysis of blood, samples were assessed immediately after extraction using an impedance-based hematology analyser (Mythic 18, Woodley Veterinary Diagnostics; ref. 34). The output parameter studied was hemoglobin concentration (Hb, g/dL). EDTA-treated blood samples were then centrifuged at 4,500 × *g* for 10 minutes, the supernatants were aliquoted and kept at −80°C until further use. For mouse and human VEGF immunodetection (mVEGF and hVEGF, respectively) Quantikine ELISA kits (R&D) were used. Plasma samples were diluted 1:10 and 50 µL per well was used to quantify the concentration. For Erythropoietin EPO DuoSet ELISA (R&D) was used at a dilution 1:4. For the three ELISA kits, procedure was carried out according to the manufacturer instructions.

### Image analysis

PAT data analysis was performed using ViewMSOT software (v3.6.0.119; iThera Medical GmbH). Model-based image reconstruction and linear multispectral processing were applied on data in the 700–900 nm wavelength range to retrieve the relative signal contributions of oxy (HbO_2_) and deoxy (Hb) hemoglobin. Regions of interest (ROI) were drawn manually for the tomographic section in which the tumor presented the largest area. Reference values were extracted from an ROI drawn around the abdominal aorta and vena cava in a plane taken in the same position of the oral-caudal axis for all the imaging sessions over time, before they branch for the junction with the iliac bone. ROIs were drawn over the whole tumor cross-section, in the multiwavelength view and no area within the tumor was excluded. The mean HbO_2_ and Hb intensity values in arbitrary units were extracted from the entire tumor area. PAT is only able to accurately resolve absolute SO_2_ if the recorded signal is directly related to the absorbed optical energy distribution, which requires knowledge of the light fluence distribution, system response, and Grueneisen parameter (35). We therefore denote the oxygenation metric derived in this study as an apparent metric, SO_2_^MSOT^ rather than absolute SO_2_. SO_2_^MSOT^ was computed as the ratio of the HbO_2_ signal to the total hemoglobin signal in the ROI (THb = HbO_2_+Hb). THb values were output as arbitrary units (a.u.) from the software for both tumor and reference values. To account for biological variations over time (Hb, hematocrit, and oxygenation levels) and temporal variations in system output, tumor THb values were normalized to reference values for longitudinal analyses.

Histopathologic analysis was performed on images of the entire tumor section scanned at 20× magnification using an Aperio ScanScope (Leica Biosystem). ROIs were drawn manually to delineate the viable tumor area and analyzed in Halo (v.3.0.311.293, Indica Labs). The percentage of necrotic area compared with the entire tumor area was calculated from H&E sections. Staining positivity for ASMA, F4/80, VEGF, and CA-IX was normalized to the ROI area to give a percentage positive area measurement for each stain. CD31 MVD was evaluated in CD31 sections as the ratio of the number of vessels marked by CD31 to the ROI area. The coverage of CD31-positive vessels by ASMA stain was evaluated in consecutive sections as a percentage. Mast cell density was evaluated in toluidine blue stained sections by taking the ratio of the number of positive objects to the tissue ROI area in mm^2^. No spatial correlation was performed between PAI and histology data, comparisons are made on a per-tumor basis only.

### Statistical analyses

A sample size calculation (considering *R* = 2,500 Monte Carlo samples) based on the effect sizes and within-mouse dependence level noted in our prior study (29) found that the targeted difference in SO_2_^MSOT^ means between groups would be detected with a high probability (>0.8) with a sample size of 12 tumors (i.e., 6 mice) per group. Supplementary Tables S1–S3 show the final animal and tumor numbers used.

Statistical analysis was performed using Prism (GraphPad) and R. All data are shown as mean ± SD unless otherwise stated. Significance was assigned for *P* values <0.05. Pearson product moment correlation coefficients were used to perform tests of correlations between the PAT measurements of SO_2_^MSOT^ and THb in each of the *ex vivo* IHC analyses. Log-rank tests were performed to assess significance in survival analyses. For endpoint comparisons between the groups, unpaired two-sided Student *t* test were performed, unless the data were found to have unequal variances, in which case a Welch *t* test was performed. For before and after treatment comparison of the same tumor, paired two-sided Student *t* test were performed.

For identification of the response subgroups, mouse and tumor samples of the treatment group were split into responders and nonresponders at the mouse and tumor levels using two approaches. Analyses were performed on the cubic root scale as model checks showed that a power law growth model was better able to linearize the relationship between volume and time, compared with an exponential growth model. First, we looked at the growth rate of each mouse or tumor (fitted by means of a linear model, R function stats::lm) and considering mice and tumors with a linear slope of less than 0.05 on the cube root scale as responder (Supplementary Fig. S1A and S1B, left). The probability of misclassification with this approach was found to increase as a function of sample size of the control group.

Therefore, to confirm the responder group allocation, an expectation-maximization algorithm was implemented to jointly model growth data at the mouse or tumor level, while iteratively allocating treated mice or tumors to the responder or nonresponder groups based on model parameter estimates. The model used was a random intercept and slope linear mixed model with mice/tumors as random effects. The maximization step was performed via maximization of the parameter log-likelihood using the R function lme4::lmer. The principle of the algorithm is that the variance of the residuals and variance of the random slopes should (i) decrease when the classification improves and (ii) increase in presence of misclassification. The sample size did not impact on the classification performance in this case and the same responder and nonresponder groups were identified (Supplementary Fig. S1A and S1B, right). The responder and nonresponder groups defined at the mouse or tumor levels were almost identical. The volume on the cubic root scale of each tumor of each mouse (of the control, and mouse-level nonresponder and responder groups; Supplementary Fig. S2) as a function of the number of days from enrollment shows only 1 mouse, BD22, that has a tumor classified differently according to both strategies.

For longitudinal analyses of tumor size, hierarchical random intercept linear mixed models with (i) tumor volume on the cubic root scale as outcome, (ii) number of days from enrollment, three-level group factor (control, nonresponder, responder) and their interaction as fixed effect predictors, (iii) mice and tumors within mice as hierarchical random effects, were used to model tumor growth while taking the within-tumor, within-mouse, and time dependence into account. Linear mixed models were fitted via restricted maximum likelihood (REML) by means of the function *lmer* of the R package *lme4* (version 1.1-23) and inference obtained by means of the package *lmerTest* (version 3.1-2). Sensitivity analyses considered (i) tumor-level response groups (instead of mouse-level ones), (ii) random intercept and slope at the mouse and/or tumor level (instead of random intercept only models), (iii) heteroscedastic mixed model with residual variance allowed to change with time from enrollment (fitted by means of the function *lme* of the R package *nlme*, version 3.1-149). These sensitivity analyses led to the same conclusions.

For longitudinal analyses of SO_2_^MSOT^ or THb, pointwise hierarchical random intercept linear mixed models with (i) SO_2_^MSOT^ or THb levels on the linear scale as outcome, (ii) number of days from enrollment, three-level group factor (control, nonresponder, responder) and their interaction as fixed effect predictors, (iii) mice and tumors within mice as hierarchical random effects, were used to model the SO_2_^MSOT^ or THb evolutions while taking the within-tumor, within-mouse, and time dependence into account. The pointwise linear mixed model took “mice” and “tumors within mice” as hierarchical random intercepts and “response group” and “time” (with interaction) as fixed effects. No significant effect was observed for THb so data are not presented for this biomarker. Model checks suggested the same SO_2_^MSOT^ growth per group during the first 3 weeks from enrollment and a change between groups afterward so that the predictor matrix was set accordingly, leading to better model checks and a better model fit according to the Akaike information criterion and Bayesian information criterion. Linear mixed models were fitted via REML by means of the function *lme* of the R package *nlme* (version 3.1–149). Sensitivity analyses considered (i) tumor-level response groups (instead of mouse-level ones), (ii) alternative fixed effect modeling. These sensitivity analyses led to the same conclusions.

### Data availability statement

Processed data associated with this article are available at: https://doi.org/10.17863/CAM.81065. Because of the large file size, raw data may be obtained by reasonable request to the corresponding author.

## Results

### Bevacizumab provides a survival benefit in a subset of MDA-MB-231 tumors

We commenced our study once tumors reached a volume of 0.5 cm^3^ and randomized mice into intermediate, control and bevacizumab-treated groups (Fig. 1A). Survival analysis (Fig. 1B) did not identify any MCF7 tumors in our cohort that responded to bevacizumab treatment (*P* = 0.55), while MDA-MB-231 tumors showed a heterogeneous response, with a few animals exhibiting a substantial survival benefit (*P* = 0.048). The tumors generated by MDA-MB-231 showed significantly higher MVD and less fibrosis at the enrollment timepoint (Supplementary Fig. S3A and S3B), both factors that according to previous reports influence susceptibility to bevacizumab (13, 36). In addition, at the same timepoint, systemic levels of VEGF produced by the tumor (hVEGF) were significantly higher in MDA-MB-231 compared with MCF7, while VEGF coming from the tumor microenvironment (mVEGF) was significantly lower (Supplementary Fig. S3C and S3D).

To separate the bevacizumab-treated MDA-MB-231 tumors according to the nature of their response, we performed linear regression modeling on the tumor volume as a function of time on a per mouse (Supplementary Fig. S1) and per tumor basis (Supplementary Fig. S2) and considered treated mice or tumors that showed a slope parameter significantly different from the control group as being responders. Final animal numbers for each group and experiment are detailed in Supplementary Tables S1–S3. The tumor survival (Supplementary Fig. S4) and growth curves (Fig. 1C) according to this grouping illustrate a significant growth inhibition in the responding group (Bev-R) compared with the nonresponding group (Bev-NR) and control (Ctrl). As expected, the nonresponder and control groups show no statistical difference in growth levels on average, while the responder group differs significantly from the control and nonresponders.

### Bevacizumab sequesters hVEGF in mice bearing responding MDA-MB-231 tumors

**Figure 2.**
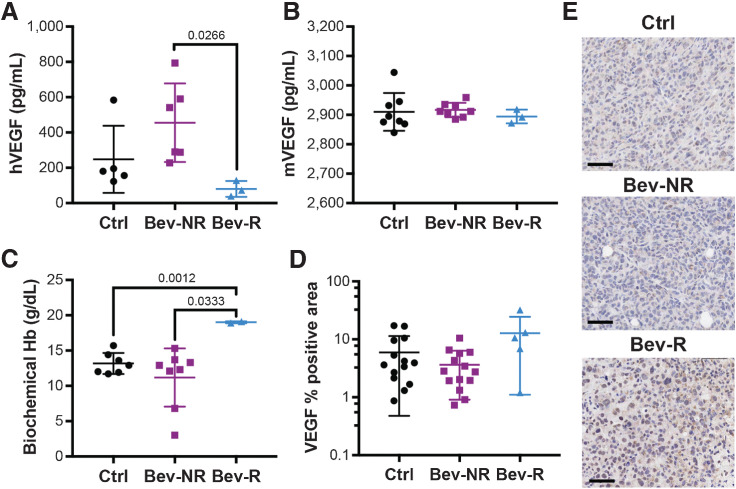
Bevacizumab treatment in MDA-MD-231 tumors changes systemic VEGF and hemoglobin levels, while influencing VEGF positivity in the responding tumors. **A** and **B,** Systemic levels of hVEGF (**A**) in the circulation are significantly reduced in responding mice but no change in mVEGF (**B**) was observed (Ctrl, *n*_mice_ = 5; Bev-NR, *n*_mice_ = 6; Bev-R, *n*_mice_ = 3). **C,** Responding mice also showed a significant increase in systemic levels of biochemical hemoglobin (Ctrl, *n*_mice_ = 7; Bev-NR, *n*_mice_ = 8; Bev-R, *n*_mice_ = 2). **D** and **E,** hVEGF measured using IHC in the respective tumors showed a significant increase in positivity in the responders (Ctrl, *n*_tumors_ = 14, Bev-NR, *n*_tumors_ = 14; Bev-R, *n*_tumors_ = 5). Scale bars, 50 µm. *P* values are displayed from two-sided Student *t* tests except in **D**, where a Welch *t* test was performed because of unequal variances.

To confirm and understand the impact of the bevacizumab therapy on MDA-MB-231 tumors, we analyzed the concentrations of hVEGF and mVEGF in the different groups. The concentration of hVEGF in the Bev-R group was significantly lower than in the Bev-NR group at the final timepoint (Fig. 2A; 80 ± 26 pg/mL vs. 455 ± 91 pg/mL), suggesting that bevacizumab is effective at sequestering VEGF in these mice. We noted that the resistant MCF7 tumors increased hVEGF in response to Bev therapy (Supplementary Fig. S5A) to levels analogous to the Bev-NR (497.2 ± 106.9 and 455 ± 91 pg/mL, respectively). This might indicate that bevacizumab fails to sequester hVEGF from MCF7 and Bev-NR MDA-MB-231 tumors. There was no significant difference in mVEGF between any groups (Fig. 2B), suggesting resistance to bevacizumab is derived from the tumor and not the mouse host. Bev-NR showed a slight elevation in hVEGF compared with Ctrl (248 ± 85 pg/mL), but this was not significant. A significant elevation of the biochemical hemoglobin was observed in the Bev-R group compared with both the Bev-NR and Ctrl groups (Fig. 2C; 19 ± 0.1 g/dL vs. 11.1 ± 1.5 g/dL and 13.1 ± 0.6 g/dL, respectively); however, no significant changes in erythropoietin were observed in these mice either in absolute terms (Ctrl = 0.47 ± 0.19; Bev-NR = 1.32 ± 0.47; Bev-R = 0.97 ± 0.50 mIU/mL) or relative to tumor volume (Ctrl = 0.38 ± 0.18; Bev-NR = 0.59 ± 0.27; Bev-R = 6.23 ± 5.91 mIU/mL * cm^3^_TUM_). Staining for VEGF within the tumor tissue (Fig. 2D and E) tended toward an increase in positive area in the Bev-R tumors compared with Bev-NR tumors (12.8% ± 5.2% vs. 3.6% ± 0.7%), although this was not significant. Our results may indicate a compensatory response in the tumor tissue to the blockade of systemic VEGF.

### PAT reveals differences in oxygenation and total hemoglobin content between bevacizumab responders and nonresponders

**Figure 3.**
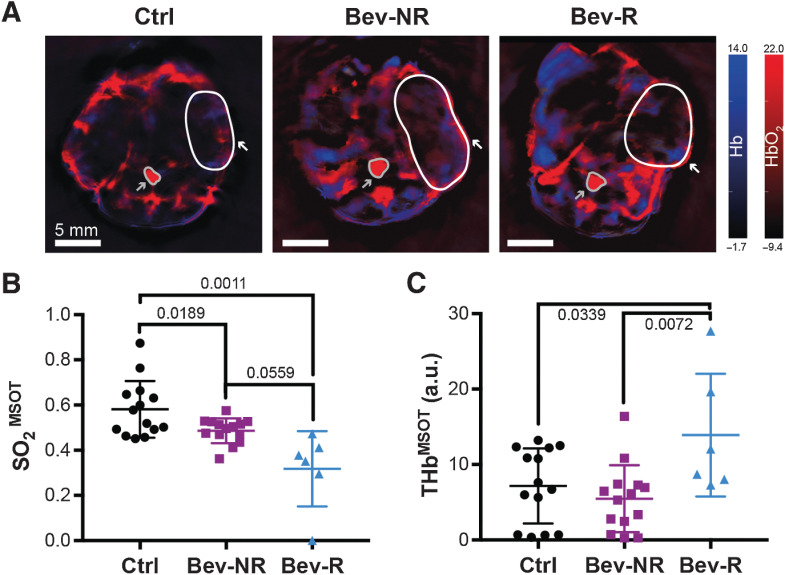
PAT detects changes in tumor vasculature in response to bevacizumab treatment. **A,** Representative PAT images at endpoint denoting deoxyhemoglobin (Hb) on the blue color scale and oxyhemoglobin (HbO_2_) on the red color scale. Regions of interest shown denote the position of one tumor per mouse (white outline and arrow) and the aorta/vena cava (gray outline and arrow), used as a reference. **B,** Tumor oxygenation extracted from PAT images (SO_2_^MSOT^) showed a significant decrease in both Bev-NR and Bev-R groups compared with Ctrl. The Bev-R group also had significantly lower oxygenation than the Bev-NR group. **C,** Hemoglobin content extracted from PAT images (THb^MSOT^) was significantly elevated in the Bev-R group compared with both Bev-NR and Ctrl. Ctrl, *n*_tumors_ = 14; Bev-NR, *n*_tumors_ = 14; Bev-R, *n*_tumors_ = 6. Scale bars, 5 mm. *P* values are displayed from two-sided Welch (**B**) and Student (**C**) *t* tests.

We first analyzed the imaging data obtained at endpoint in the MCF7 model and confirmed that no significant differences in PAT data were observed between treated and control tumors (Supplementary Fig. S5B). Analogously, THb in MDA-MB-231 cohort also showed no differences overall between the treated and control tumors. Interestingly, however, the Bev-R group showed a significant elevation of their THb content compared with both Bev-NR and Ctrl groups (Fig. 3C, 13.9 ± 3.3 a.u. vs. 5.5 ± 1.2 a.u. and 7.2 ± 1.3 a.u.). We therefore concentrated the remainder of the study on comparing our findings across the MDA-MB-231 tumors according to the Ctrl, Bev-NR, and Bev-R groupings.

Visualization of the HbO_2_- and Hb-weighted photoacoustic signals in tumors at endpoint (Fig. 3A) showed an overall enhancement of Hb-weighted signal in the bevacizumab-treated tumors, which upon quantification, translated into a decrease in SO_2_^MSOT^ (Fig. 3B). Notably, both the Bev-NR and Bev-R groups showed decreased SO_2_^MSOT^ relative to Ctrl, though the effect was larger in the Bev-R tumors (0.48 ± 0.01 and 0.31 ± 0.07 vs. 0.58 ± 0.03). SO_2_^MSOT^ appears to be a more sensitive metric than THb, as it shows a treatment effect on the tumor oxygenation even without impact in the tumor volume.

### Oxygen saturation measured using PAT is indicative of the observed survival benefit in responders

**Figure 4.**
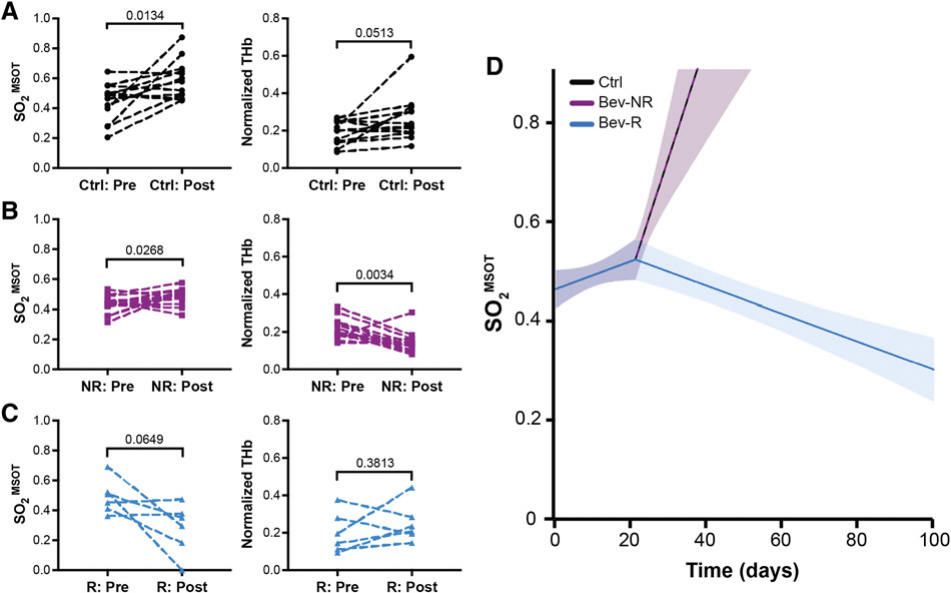
Longitudinal analysis demonstrates that PAT can be used to indicate survival benefit from bevacizumab therapy. **A** and **B,** Oxygenation (SO_2_^MSOT^) and normalized hemoglobin content (THb) increase from enrollment (noted as “pre”) to endpoint of tumor excision (noted as “post”) in the Ctrl group (**A**), while in the Bev-NR group (**B**), SO_2_^MSOT^ increases concurrently with a decrease in normalized THb. **C,** Conversely, the Bev-R group trend toward a significant decrease in SO_2_^MSOT^, with no significant change in normalized THb observed. **D,** Estimated SO_2_^MSOT^ level as a function of time (number of days from enrollment) and group (color). Trendlines are shown alone for clarity; individual tumor trajectories are shown in Supplementary Fig. S5. Ctrl tumor data parallel Bev-NR data. Analysis of SO_2_^MSOT^ over time during the study showed a significant (*P* < 0.0001) change in slope at 3 weeks after enrollment in the Bev-R group compared with either of the Ctrl or Bev-NR groups. Ctrl, *n*_tumors_ = 14 and *n*_mice_ = 8; Bev-NR, *n*_tumors_ = 14 and *n*_mice_ = 8; Bev-R, *n*_tumors_ = 6 and *n*_mice_ = 3. *P* values are displayed from two-sided paired Student *t* tests; shaded areas in **D** denote the 95% pointwise confidence bounds for the average.

Having established the ability of PAT to resolve differences between the responding and nonresponding groups at endpoint, we then considered whether PAT could be applied to predict the status of these tumors earlier in the treatment time course. Initially, we compared data acquired at the point of enrollment with that at the endpoint. These data indicated that both SO_2_^MSOT^ and normalized THb increase over the time course in Ctrl group (Fig. 4A). The Bev-NR group paralleled the Ctrl group with an increase in SO_2_^MSOT^ but conversely showed a significant decreased in normalized THb (Fig. 4B). The Bev-R group, on the other hand, showed a trend toward decreased SO_2_^MSOT^ but no significant change in normalized THb over the time course (Fig. 4C). Next, longitudinal weekly imaging data were fitted with a pointwise linear mixed model (Fig. 4D; Supplementary Fig. S6), which showed a change in slope at 3 weeks after enrollment. Performing this specific modeling of SO_2_^MSOT^ as a function of response group confirms that there is a clear association between SO_2_^MSOT^ and outcome that can be detected by PAT already at 3 weeks after enrollment. No such relationship could be identified using THb measurements, suggesting that SO_2_^MSOT^, which reflects tumor vascular function (25, 37) and is a generally more robust PAT biomarker (33), is a better indicator of outcome than THb, which reflects purely hemoglobin content.

### Significant changes in photoacoustic imaging biomarkers reflect underlying biological differences in the responders and nonresponders

**Figure 5.**
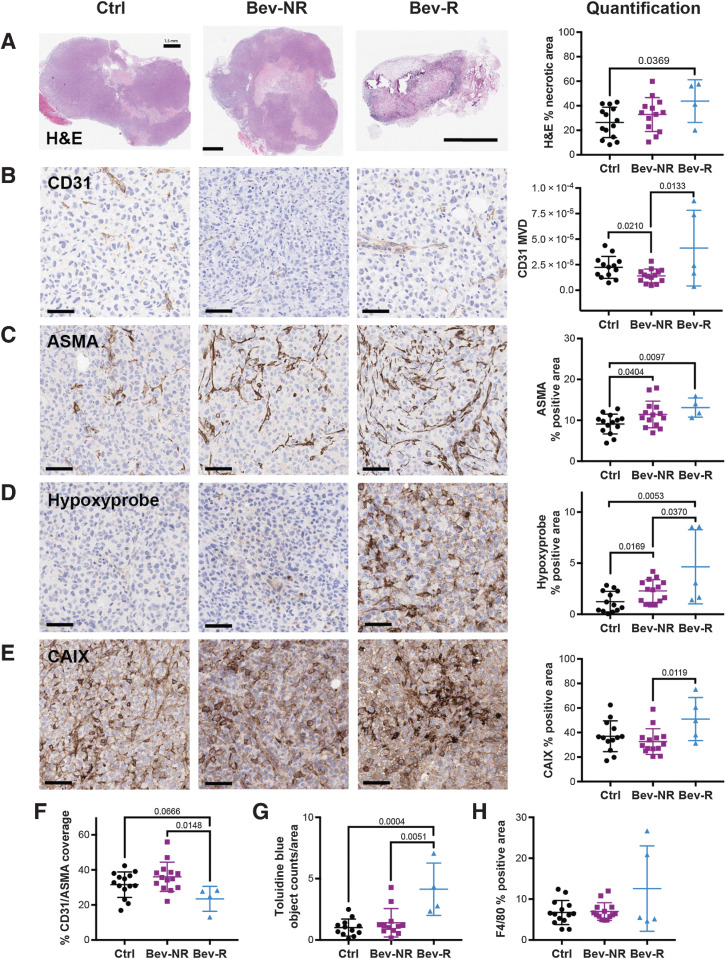
*Ex vivo* histopathologic analysis of the tumor vascular microenvironment explains the PAT findings. **A,** H&E staining. Scale bar, 1.5 mm. Necrosis was significantly higher in the Bev-R group compared with Ctrl (Ctrl, *n*_tumors_ = 14; Bev-NR, *n*_tumors_ = 13; Bev-R, *n*_tumors_ = 4). **B,** CD31-positive MVD (vessel/μm^2^) was significantly higher for the Bev-R group compared with the Bev-NR group, which itself was lower than Ctrl (Ctrl, *n*_tumors_ = 14; Bev-NR, *n*_tumors_ = 13; Bev-R, *n*_tumors_ = 5). **C,** ASMA positivity was significantly higher in both the Bev-NR and Bev-R groups compared with Ctrl (Ctrl, *n*_tumors_ = 14; Bev-NR, *n*_tumors_ = 14; Bev-R, *n*_tumors_ = 4). **D,** Hypoxyprobe, denoting areas of hypoxia-dependent pimonidazole labeling in the tumor tissue, was significantly higher for the Bev-R group compared with the Bev-NR group, which itself was higher than Ctrl (Ctrl, *n*_tumors_ = 13; Bev-NR, *n*_tumors_ = 14; Bev-R, *n*_tumors_ = 5). **E,** CAIX positivity, which also reflects tissue hypoxia, was significantly increased in the Bev-R group compared with the Bev-NR group (Ctrl, *n*_tumors_ = 13; Bev-NR, *n*_tumors_ = 14; Bev-R, *n*_tumors_ = 5). **F,** Coverage of CD31-positive vessels by ASMA staining was significantly lower in the Bev-R group compared with Bev-NR and also lower than Ctrl (Ctrl, *n*_tumors_ = 14; Bev-NR, *n*_tumors_ = 14; Bev-R, *n*_tumors_ = 4). **G** and **H,** Toluidine blue–positive objects per mm^2^, reflective of mast cell density in the tumor, were significantly higher in Bev-NR compared with Bev-R and Ctrl groups (Ctrl, *n*_tumors_ = 12; Bev-NR, *n*_tumors_ = 12; Bev-R, *n*_tumors_ = 4; **G**), though no significant differences in F4/80 staining (**H**), reflecting macrophages, was observed (Ctrl, *n*_tumors_ = 14; Bev-NR, *n*_tumors_ = 14; Bev-R, *n*_tumors_ = 5). Scale bars, 50 µm. *P* values are displayed from two-sided Student *t* tests.

Finally, we sought to confirm the changes in the tumor vascular phenotype that give rise to the differences observed in our PAT data by performing IHC analysis of tumors at the endpoint. As could be expected, Bev-R tumors showed a significant increase in necrosis compared with Ctrl (Fig. 5A; 44% ± 9% vs. 26% ± 3%). The treatment had some marginal vascular effect in Bev-NR as CD31-positive MVD was lower in the Bev-NR group compared with Ctrl (Fig. 5B; 1.4 ± 0.2 × 10^−5^ vs. 2.2 ± 0.3 × 10^−5^). Conversely, MVD was higher in the Bev-R compared with the Bev-NR group (4.1 ± 1.7 × 10^−5^ vs. 1.4 ± 0.2 × 10^−5^).

Both Bev-NR and Bev-R tumors showed a small but significant increase in ASMA staining (Fig. 5C) compared with Ctrl (11.4% ± 0.9% and 13.1% ± 1.2% vs. 9.1% ± 0.6%). Hypoxia appears to be elevated in the Bev-R group compared with Bev-NR and Ctrl, as indicated by both hypoxyprobe (Fig. 5D; 4.7% ± 1.6% vs. 2.3% ± 0.3% and 1.2% ± 0.3%) and CAIX staining (Fig. 5E; 51.0% ± 7.9% vs. 32.6% ± 2.8% and 37.0% ± 3.5%). Furthermore, colocalization of ASMA and CD31 staining indicated a significant decrease in vascular maturity in the Bev-R group compared with Bev-NR and Ctrl (Fig. 5F; 23.5% ± 3.6% vs. 36.1% ± 2.2% and 31.6% ± 1.9%), which is consistent with elevation of hypoxia despite higher overall MVD. An increase in mast cells, which can importantly affect blood vessel permeability, was also noted in Bev-R tumors (Fig. 5G). No significant change in macrophages or other white blood cells could be detected using F4/80 staining (Fig. 5H).

We finally tested for direct correlations between PAT and IHC parameters independent of treatment group. We were able to confirm significant positive correlations between THb and MVD (Supplementary Fig. S7A), as might be expected for in the case of tumor blood vessels containing more blood, as well as VEGF positive area (Supplementary Fig. S7B), suggesting more angiogenic MDA-MB-231 tumors yield a higher blood content in their vascular network (MVD and VEGF were themselves significantly correlated; *r* = 0.60, *P* < 0.05). Significant negative correlations were observed between SO_2_^MSOT^ and necrotic area (Supplementary Fig. S7C) and hypoxyprobe positivity (Supplementary Fig. S7D), confirming previous findings that measurement of SO_2_^MSOT^ with PAT relates to both necrosis and hypoxia (23, 37).

## Discussion

Despite the association between angiogenesis and patient prognosis in many solid tumors, including breast cancer, antiangiogenic therapy has failed to achieve overall survival benefits in patients. Nonetheless, bevacizumab remains approved by the EMA for use as a combination therapy to extend progression-free survival in breast cancer. Key factors for consideration in improving the outcomes of such therapy are preselection of responders and close monitoring for the onset of the failure of the therapy. Identification of predictive biomarkers to select those patients in which the therapy is likely to be efficacious and subsequently to guide dosing regimens is vital for the next generation of clinical studies with these compounds, particularly in earlier stage disease. We hypothesized that PAT biomarkers could be used to identify response and resistance to antiangiogenic therapy.

To test our hypothesis, we studied changes in oxygenation and hemoglobin content longitudinally in two mouse models undergoing bevacizumab treatment. We selected the MCF7 and MDA-MB-231 human cell line models to generate tumor xenografts in mice, as prior studies had indicated that MCF7 tumors are refractory to bevacizumab (38, 39), while MDA-MB-231 tumors are sensitive (5, 40, 41). At the time of enrollment for treatment, there were substantial differences between the models, with MDA-MD-231 showing higher MVD and a lower collagen content in extracellular matrix (13). This aligns with previous results showing that higher MVD pretreatment indicates better response to bevacizumab, while fibrosis may impede therapy response (36). Aligned with these prior studies, we found a survival benefit only in our MDA-MB-231 cohort but not in the MCF7 cohort. Interestingly, the response of our MDA-MB-231 tumors was more heterogeneous than in previous studies, with both a responding and nonresponding group emerging. This apparent deviation from prior reports is likely due to the disparity in treatment regimens between prior studies, where more acute and frequent dosing was used; we aimed to mimic a more clinically relevant application of the drug (42), with a longer-term lower-frequency dosing akin to a metronomic approach (43).

Circulating levels of VEGF provided us with useful insights. First, the MCF7 cohort produced high levels of host murine, while the partially sensitive MDA-MB-231 cohort produced VEGF mainly from the tumor cells (human) source. Second, circulating hVEGF levels were lower in the MDA-MB-231 Bev-R group compared with Bev-NR, yet were elevated in both the resistant MCF7 cohort and Bev-NR MDA-MB-231 group relative to their respective controls. The fall in circulating hVEGF in the Bev-R group occurred in the context of a concurrent increase in VEGF staining in those tumors, which may reflect an on-target effect of the treatment whereby the tumor is compensating for bevacizumab therapy by increasing VEGF, but could also be explained by the broader hypoxia response that would stimulate further expression of VEGF.

Our *in vivo* imaging findings confirm our hypothesis that PAT is able to identify response and resistance to antiangiogenic therapy in our animal cohorts. In MDA-MB-231, SO_2_^MSOT^ seems a more sensitive metric than THb^MSOT^, as it is able to detect the subtle changes in the vascular and hypoxia phenotype of the Bev-NR group that are seen in the histologic analysis. Notably, increases in biochemical Hb may be reflected in THb^MSOT^, which merits longitudinal analysis in future studies. Moreover, we were able to show that measurements of tumor oxygenation, denoted by SO_2_^MSOT^, diverge in the responding group within the first 3 weeks of treatment and show sustained differences throughout the time course until endpoint, indicating potential for PAT to predict survival benefit at an early stage in the treatment time course. These findings parallel the time course of changes in tumor volume, and are consistent with other recent reports on the use of PAT in monitoring bevacizumab response (23). Importantly, the use of PAT biomarkers enables direct confirmation of the impact of the therapy on the tumor vasculature, which is important from a clinical perspective given the common emergence of resistance to such therapies. Knowledge of the vascular maturity and oxygenation dynamics could be furthered by applying a gas challenge, which has been shown in mouse models to provide surrogate biomarkers of hypoxia and necrosis (37). It was not applied in the current study as the tumor models used already exhibit striking differences in terms of static PAT metrics (29) and the application of the gas challenge extends the animal procedure time, which was considered inappropriate for animal welfare in this longitudinal study over many weeks with repeated imaging sessions.

The observations made from our *in vivo* data were then confirmed using IHC analyses *ex vivo*. Taken together, our IHC data indicate that response to bevacizumab treatment is heterogenous, influencing features of hypoxia and angiogenesis, and ultimately impacting tumor volume response and survival. In MDA-MB-231 Bev-R tumors show an overall higher vessel density, which could be a result of their elevated VEGF expression in the tumor tissue, but the resulting angiogenic vasculature is less mature and results in higher hypoxia and cell death. Conversely, Bev-NR tumors show a general decrease in MVD, which may instead cause only transitory hypoxia, as no change is observed in long-term markers such as CA-IX or VEGF but is indicated with hypoxyprobe staining. Bev-NR also showed a significant increase in vascular normalization, marked by CD31/ASMA, suggesting the possibility that when bevacizumab treatment alone does not produce long-term hypoxia, it cannot generate a response in terms of tumor volume change or survival. These findings are consistent with a prior report of successful bevacizumab treatment in MDA-MB-231 tumors (41).

Despite our promising findings, there remain some limitations to our study. First, the orthotopic implantation of our tumors fails to recapitulate the advanced metastatic disease in which antiangiogenic therapy is often tested and applied (44). Transplantation models may also generate an overly angiogenic phenotype. Our findings should therefore be validated in other relevant models before drawing conclusions as to the utility of PAT in predicting survival benefit with antiangiogenic therapy. Second, we used only a single-agent treatment with bevacizumab; further work is needed to evaluate whether our findings could be extrapolated to the case of combination therapy, which is the current clinical-use case. Third, we tested a long-term low-dose regimen in our study, akin to the clinical setting, and found a diversity of response, which was not observed in other studies using higher and/or more frequent doses (40, 45–47). Performing PAT under a range of dosing regimens could evaluate the potential of PAT biomarkers to identify the most effective dose to elicit survival benefit and also test their behavior upon emergence of resistance, which is often observed preclinically after an initial treatment response (38). Finally, there are some limitations pertaining to the statistical analysis of our data. Our final sample sizes per group were modest meaning that, even though the effect size was expected to be large, some analyses may be underpowered. In such cases, while the type I error per analysis would still be close to 5%, the estimated differences of interest would likely be overestimated. Furthermore, to optimize the statistical power, we apply several statistical analyses mostly without multiplicity adjustment, meaning that the overall type I error rate (familywise error rate) is likely above 5%. Also, our algorithm for assigning response groups had a lower specificity (∼90%) compared with sensitivity (>95%) when the average difference of growth rate between groups is small.

In summary, we have demonstrated the potential for PAT to indicate response or resistance to antiangiogenic therapy in a longitudinal setting. PAT could thus find application in preclinical studies evaluating antiangiogenic therapy combinations, particularly with metronomic chemotherapy where there is expected to be synergistic antiangiogenic effects. The tomographic approach used here could be applied to more deep-seated tumors, including the use of transgenic models to study tumors that better recapitulate the heterogeneity observed in patients. PAT has also been tested in the clinic for the staging of suspicious breast lesions. Upon further validation of our findings, it may be interest to evaluate the clinical value of PAT biomarkers in a treatment response setting.

## Supplementary Material

Supplementary Data

## References

[1] Michiels C , Tellier C, Feron O. Cycling hypoxia: a key feature of the tumor microenvironment. Biochim Biophys Acta 2016;1866:76–86. 27343712 10.1016/j.bbcan.2016.06.004

[2] Gillies RJ , Brown JS, Anderson ARA, Gatenby RA. Eco-evolutionary causes and consequences of temporal changes in intratumoural blood flow. Nat Rev Cancer 2018;18:576–85. 29891961 10.1038/s41568-018-0030-7 PMC6441333

[3] Hanahan D , Weinberg RA. Hallmarks of cancer: the next generation. Cell 2011;144:646–74. 21376230 10.1016/j.cell.2011.02.013

[4] Bergers G , Hanahan D. Modes of resistance to anti-angiogenic therapy. Nat Rev Cancer 2008;8:592–603. 18650835 10.1038/nrc2442 PMC2874834

[5] Kazmierczak PM , Schneider M, Habereder T, Hirner-Eppeneder H, Eschbach RS, Moser M, . αvß3-integrin–targeted magnetic resonance imaging for the assessment of early antiangiogenic therapy effects in orthotopic breast cancer xenografts. Invest Radiol 2016;51:746–55. 27082316 10.1097/RLI.0000000000000278

[6] Madu CO , Wang S, Madu CO, Lu Y. Angiogenesis in breast cancer progression, diagnosis, and treatment. J Cancer 2020;11:4474–94. 32489466 10.7150/jca.44313 PMC7255381

[7] Gray R , Bhattacharya S, Bowden C, Miller K, Comis RL. Independent review of E2100: a phase III trial of bevacizumab plus paclitaxel versus paclitaxel in women with metastatic breast cancer. J Clin Oncol 2009;27:4966–72. 19720913 10.1200/JCO.2008.21.6630 PMC2799052

[8] Gonzalez-Angulo AM , Hortobagyi GN, Ellis LM. Targeted therapies: Peaking beneath the surface of recent bevacizumab trials. Nat Rev Clin Oncol 2011;8:319–20. 21556024 10.1038/nrclinonc.2011.66

[9] Aalders KC , Tryfonidis K, Senkus E, Cardoso F. Anti-angiogenic treatment in breast cancer: facts, successes, failures and future perspectives. Cancer Treat Rev 2017;53:98–110. 28088074 10.1016/j.ctrv.2016.12.009

[10] Morotti M , Dass PH, Harris AL, Lord S. Pharmacodynamic and pharmacokinetic markers for anti-angiogenic cancer therapy: implications for dosing and selection of patients. Eur J Drug Metab Pharmacokinet 2018;43:137–53. 29019020 10.1007/s13318-017-0442-x

[11] Kerbel RS , Kamen BA. The anti-angiogenic basis of metronomic chemotherapy. Nat Rev Cancer 2004;4:423–36. 15170445 10.1038/nrc1369

[12] Curtis C , Shah SP, Chin S-F, Turashvili G, Rueda OM, Dunning MJ, . The genomic and transcriptomic architecture of 2,000 breast tumours reveals novel subgroups. Nature 2012;486:346–52. 22522925 10.1038/nature10983 PMC3440846

[13] Tolaney SM , Boucher Y, Duda DG, Martin JD, Seano G, Ancukiewicz M, . Role of vascular density and normalization in response to neoadjuvant bevacizumab and chemotherapy in breast cancer patients. Proc Natl Acad Sci U S A 2015;112:14325–30. 26578779 10.1073/pnas.1518808112 PMC4655544

[14] Vasudev NS , Reynolds AR. Anti-angiogenic therapy for cancer: current progress, unresolved questions and future directions. Angiogenesis 2014;17:471–94. 24482243 10.1007/s10456-014-9420-y PMC4061466

[15] Kuczynski EA , Vermeulen PB, Pezzella F, Kerbel RS, Reynolds AR. Vessel co-option in cancer. Nat Rev Clin Oncol 2019;16:469–93. 30816337 10.1038/s41571-019-0181-9

[16] Murphy P , Koh DM. Imaging in clinical trials. Cancer Imaging 2010;10:S74–82. 20880784 10.1102/1470-7330.2010.9027 PMC2967134

[17] Manohar S , Dantuma M. Current and future trends in photoacoustic breast imaging. Photoacoustics 2019;16:100134. 31871887 10.1016/j.pacs.2019.04.004 PMC6909206

[18] Brown E , Brunker J, Bohndiek SE. Photoacoustic imaging as a tool to probe the tumour microenvironment. Dis Model Mech 2019;12:dmm039636. 31337635 10.1242/dmm.039636 PMC6679374

[19] May JP , Hysi E, Wirtzfeld LA, Undzys E, Li SD, Kolios MC. Photoacoustic imaging of cancer treatment response: early detection of therapeutic effect from thermosensitive liposomes. PLoS One 2016;11:e0165345. 27788199 10.1371/journal.pone.0165345 PMC5082794

[20] Mallidi S , Watanabe K, Timerman D, Schoenfeld D, Hasan T. Prediction of tumor recurrence and therapy monitoring using ultrasound-guided photoacoustic imaging. Theranostics 2015;5:289–301. 25553116 10.7150/thno.10155 PMC4279192

[21] Ghosh P , Guo Y, Ashrafi A, Chen J, Dey S, Zhong S, . Oxygen-enhanced optoacoustic tomography reveals the effectiveness of targeting heme and oxidative phosphorylation at normalizing tumor vascular oxygenation. Cancer Res 2020;80:3542–55. 32546631 10.1158/0008-5472.CAN-19-3247 PMC7721224

[22] Zhou H-C , Chen N, Zhao H, Yin T, Zhang J, Zheng W, . Optical-resolution photoacoustic microscopy for monitoring vascular normalization during anti-angiogenic therapy. Photoacoustics 2019;15:100143. 31463195 10.1016/j.pacs.2019.100143 PMC6710376

[23] Liapis E , Klemm U, Karlas A, Reber J, Ntziachristos V. Resolution of spatial and temporal heterogeneity in bevacizumab-treated breast tumors by eigenspectra multispectral optoacoustic tomography. Cancer Res 2020;80:5291–5304. 32994204 10.1158/0008-5472.CAN-20-1011

[24] Pham E , Yin M, Peters CG, Lee CR, Brown D, Xu P, . Preclinical efficacy of bevacizumab with CRLX101, an investigational nanoparticle-drug conjugate, in treatment of metastatic triple-negative breast cancer. Cancer Res 2016;76:4493–503. 27325647 10.1158/0008-5472.CAN-15-3435

[25] Bohndiek SE , Sasportas LS, Machtaler S, Jokerst JV, Hori S, Gambhir SS. Photoacoustic tomography detects early vessel regression and normalization during ovarian tumor response to the antiangiogenic therapy trebananib. J Nucl Med 2015;56:1942–7. 26315834 10.2967/jnumed.115.160002 PMC5612481

[26] Yang J , Zhang G, Li Q, Liao C, Huang L, Ke T, . Photoacoustic imaging for the evaluation of early tumor response to antivascular treatment. Quant Imaging Med Surg 2019;9:160–70. 30976540 10.21037/qims.2018.11.06 PMC6414773

[27] Okumura K , Yoshida K, Yoshioka K, Aki S, Yoneda N, Inoue D, . Photoacoustic imaging of tumour vascular permeability with indocyanine green in a mouse model. Eur Radiol Exp 2018;2:5. 29708213 10.1186/s41747-018-0036-7 PMC5909364

[28] Longo DL , Stefania R, Callari C, De Rose F, Rolle R, Conti L, . Water soluble melanin derivatives for dynamic contrast enhanced photoacoustic imaging of tumor vasculature and response to antiangiogenic therapy. Adv Healthc Mater 2017;6. 10.1002/adhm.201600550 27782375

[29] Quiros-Gonzalez I , Tomaszewski MR, Aitken SJ, Ansel-Bollepalli L, McDuffus L-A, Gill M, . Optoacoustics delineates murine breast cancer models displaying angiogenesis and vascular mimicry. Br J Cancer 2018;118:1098–106. 29576623 10.1038/s41416-018-0033-x PMC5931091

[30] Laufer JG , Zhang EZ, Treeby BE, Cox BT, Beard PC, Johnson P, . *In vivo* preclinical photoacoustic imaging of tumor vasculature development and therapy. J Biomed Opt 2012;17:056016. 22612139 10.1117/1.JBO.17.5.056016

[31] Dima A , Burton NC, Ntziachristos V. Multispectral optoacoustic tomography at 64, 128, and 256 channels. J Biomed Opt 2014;19:36021. 24676383 10.1117/1.JBO.19.3.036021

[32] Morscher S , Driessen WHP, Claussen J, Burton NC. Semi-quantitative multispectral optoacoustic tomography (MSOT) for volumetric PK imaging of gastric emptying. Photoacoustics 2014;2:103–10. 25431754 10.1016/j.pacs.2014.06.001 PMC4244636

[33] Joseph J , Tomaszewski MR, Quiros-Gonzalez I, Weber J, Brunker J, Bohndiek SE. Evaluation of precision in optoacoustic tomography for preclinical imaging in living subjects. J Nucl Med 2017;58:807–14. 28126890 10.2967/jnumed.116.182311

[34] Wassmuth AK , Riond B, Hofmann-Lehmann R, Lutz H. Evaluation of the Mythic 18 hematology analyzer for use with canine, feline, and equine samples. J Vet Diagn Invest 2011;23:436–53. 21908272 10.1177/1040638711403416

[35] Cox B , Laufer JG, Arridge SR, Beard PC. Quantitative spectroscopic photoacoustic imaging: a review. J Biomed Opt 2012;17:061202. 22734732 10.1117/1.JBO.17.6.061202

[36] Henke E , Nandigama R, Ergün S. Extracellular matrix in the tumor microenvironment and its impact on cancer therapy. Front Mol Biosci 2020;6:160. 32118030 10.3389/fmolb.2019.00160 PMC7025524

[37] Tomaszewski MR , Gehrung M, Joseph J, Quiros-Gonzalez I, Disselhorst JA, Bohndiek SE. Oxygen-enhanced and dynamic contrast-enhanced optoacoustic tomography provide surrogate biomarkers of tumor vascular function, hypoxia, and necrosis. Cancer Res 2018;78:5980–91. 30115696 10.1158/0008-5472.CAN-18-1033

[38] Curtarello M , Zulato E, Nardo G, Valtorta S, Guzzo G, Rossi E, . VEGF-targeted therapy stably modulates the glycolytic phenotype of tumor cells. Cancer Res 2015;75:120–33. 25381153 10.1158/0008-5472.CAN-13-2037

[39] Zhu W , Kato Y, Artemov D. Heterogeneity of tumor vasculature and antiangiogenic intervention: insights from MR angiography and DCE-MRI. PLoS One 2014;9:e86583. 24466160 10.1371/journal.pone.0086583 PMC3900564

[40] EL-Hajjar L , Jalaleddine N, Shaito A, Zibara K, Kazan JM, El-Saghir J, . Bevacizumab induces inflammation in MDA-MB-231 breast cancer cell line and in a mouse model. Cell Signal 2019;53:400–12. 30445167 10.1016/j.cellsig.2018.11.007

[41] Roland CL , Dineen SP, Lynn KD, Sullivan LA, Dellinger MT, Sadegh L, . Inhibition of vascular endothelial growth factor reduces angiogenesis and modulates immune cell infiltration of orthotopic breast cancer xenografts. Mol Cancer Ther 2009;8:1761–71. 19567820 10.1158/1535-7163.MCT-09-0280

[42] Rossari JR , Metzger-Filho O, Paesmans M, Saini KS, Gennari A, de Azambuja E, . Bevacizumab and breast cancer: a meta-analysis of first-line phase III studies and a critical reappraisal of available evidence. J Oncol 2012;2012:417673. 23008712 10.1155/2012/417673 PMC3447373

[43] Liu Y , Gu F, Liang J, Dai X, Wan C, Hong X, . The efficacy and toxicity profile of metronomic chemotherapy for metastatic breast cancer: a meta-analysis. PLoS One 2017;12:e0173693. 28296916 10.1371/journal.pone.0173693 PMC5351982

[44] Kerbel RS , Guerin E, Francia G, Xu P, Lee CR, Ebos JML, . Preclinical recapitulation of antiangiogenic drug clinical efficacies using models of early or late stage breast cancer metastatis. Breast 2013;22:S57–S65. 24074794 10.1016/j.breast.2013.07.011

[45] von Baumgarten L , Brucker D, Tirniceru A, Kienast Y, Grau S, Burgold S, . Bevacizumab has differential and dose-dependent effects on glioma blood vessels and tumor cells. Clin Cancer Res 2011;17:6192–205. 21788357 10.1158/1078-0432.CCR-10-1868

[46] Selvakumaran M , Amaravadi RK, Vasilevskaya IA, O'Dwyer PJ. Autophagy inhibition sensitizes colon cancer cells to antiangiogenic and cytotoxic therapy. Clin Cancer Res 2013;19:2995–3007. 23461901 10.1158/1078-0432.CCR-12-1542

[47] Gu S , Xue J, Xi Y, Tang R, Jin W, Chen J-J, . Evaluating the effect of avastin on breast cancer angiogenesis using synchrotron radiation. Quant Imaging Med Surg 2019;9:418–26. 31032189 10.21037/qims.2019.03.09 PMC6462576

