# Supplementary material for: Photoacoustic Tomography Detects Response and Resistance to Bevacizumab in Breast Cancer Mouse Models
Source: Cancer Res. 2022 Apr 11;82(8):1658–68. doi: 10.1158/0008-5472.CAN-21-0626 (PMC9359720; doi:10.1158/0008-5472.CAN-21-0626)
Supplement: Supplementary Data [file can-21-0626_supplementary_data_suppsd1.pdf]

Supplementary Information

Photoacoustic tomography indicates response and resistance to Bevacizumab in breast cancer mouse models.

Isabel Quiros-Gonzalez[1,2], Michal R Tomaszewski[1,2], Monika A Golinska[1,2], Emma Brown[1,2], Laura Ansel-Bollepalli[1,2], Lina Hacker[1,2], Dominique-Laurent Couturier[2], Rosa M. Sainz[3], Sarah E Bohndiek[1,2*]

[1] Department of Physics, University of Cambridge, JJ Thomson Avenue, Cambridge CB3 0HE, United Kingdom

[2] Cancer Research UK Cambridge Institute, University of Cambridge, Li Ka Shing Centre, Cambridge CB2 0RE, United Kingdom

[3] Cell Morphology and Biology Department, IUOPA and ISPA, Universidad de Oviedo, Oviedo 33006, Spain

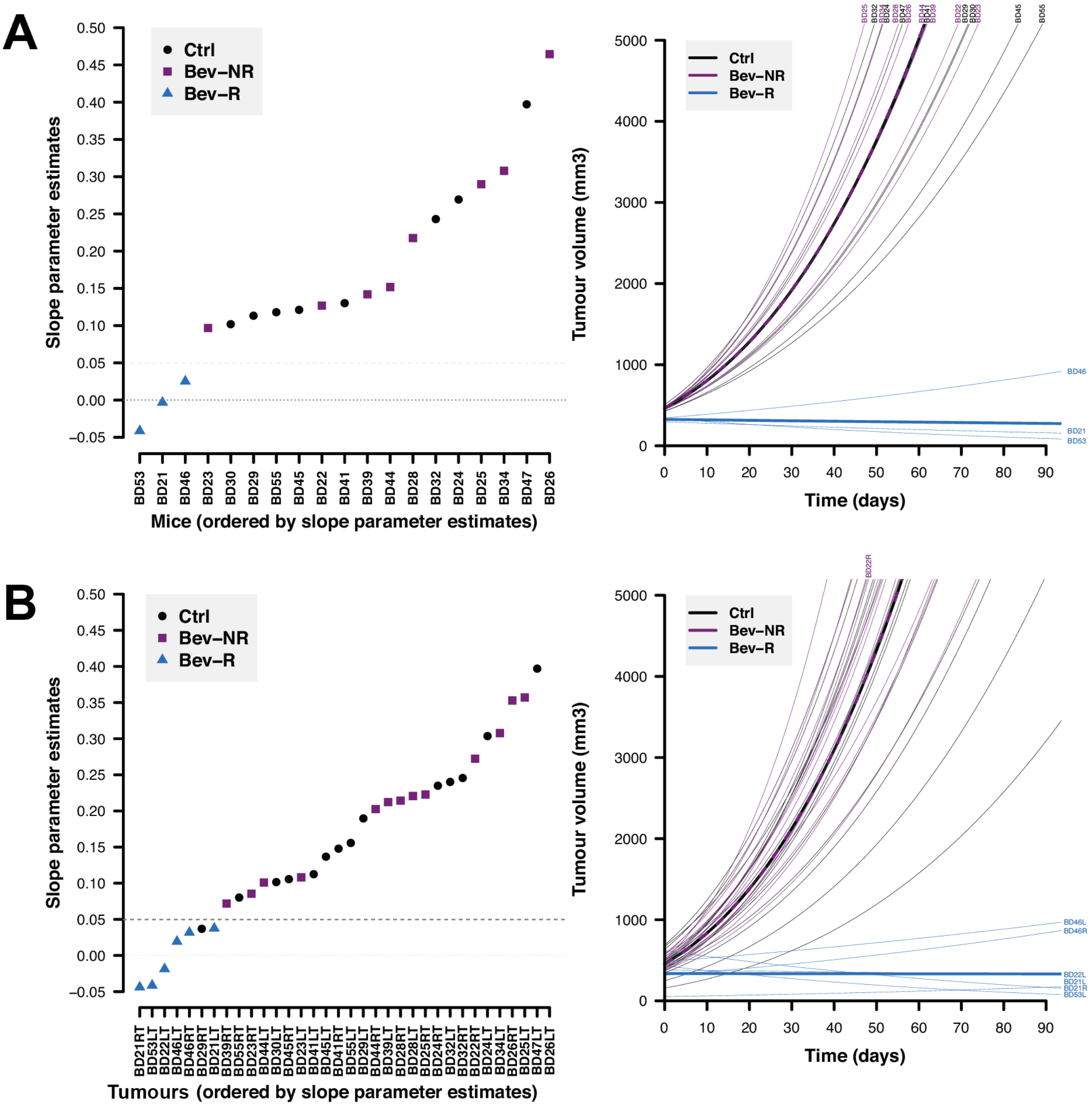

Supplementary Figure 1: Linear regression modelling enabled identification of response on a per mouse (A) and per tumour (B) basis. (A) Left: Slope parameter estimate (see Methods) on a per mouse basis, colour coded by group and ordered by estimate value, are shown for a linear regression model applied to the cube root of tumour volume. Parameters close to zero or negative mean that the tumour volume was controlled or reduced respectively. Right: the predicted responder group and predicted growth rate per mouse according to the expectation-maximisation algorithm. (B) Equivalent analysis but on a per tumour basis.

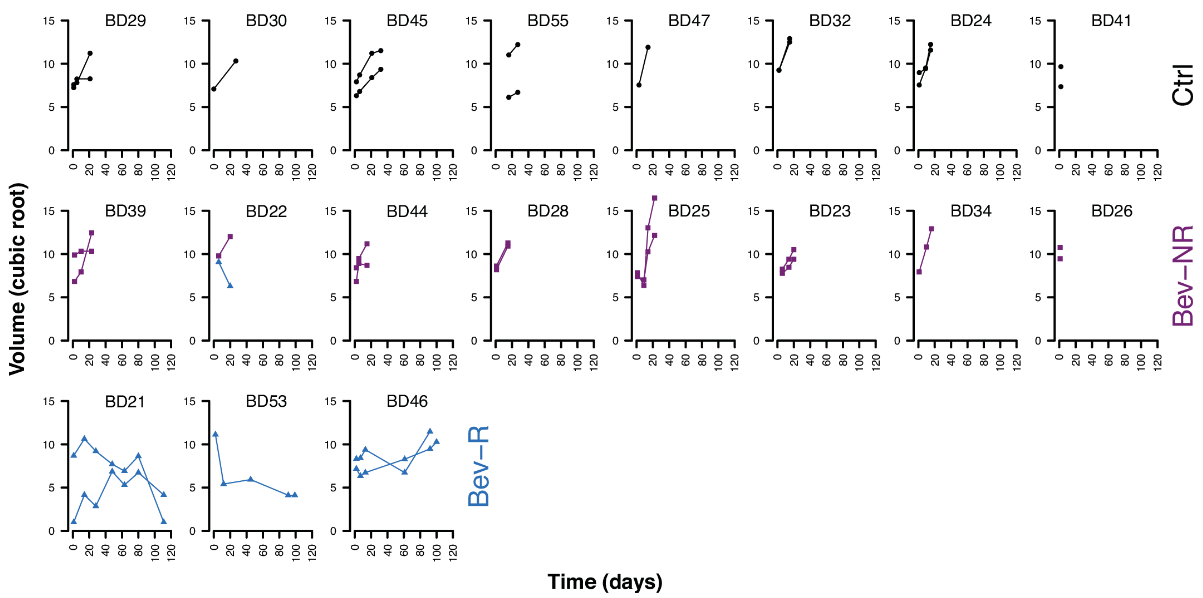

Supplementary Figure 2: Final groupings of animals and tumours by response. Volume on the cubic root scale of each tumour (lines) and each mouse (plot) grouped by mouse-level control (Ctrl), non-responding (Bev-NR) and responding (Bev-R) groups. At the mouse level, three mice showed a significant ($p<0.05$) negative difference in their slope parameter compared to the average of the control group (Supplementary Figure 1A, right) and all three animals are therefore considered to belong to the responding (Bev-R) group for the presented analyses conducted at the systemic level (i.e. serum assays). At the tumour level, 6 tumours show a significant ($p<0.05$) negative difference in their slope parameter compared to the average of the control group (Supplementary Figure 1B, right) therefore these 6 tumours are considered to belong to responding (Bev-R) group for all other analyses, which were conducted at the per tumour level. Insufficient growth rate information was available after enrolment time for BD41 or BD26 to allow growth rate modelling. In the case of BD26, neither algorithm considered it as a responder.

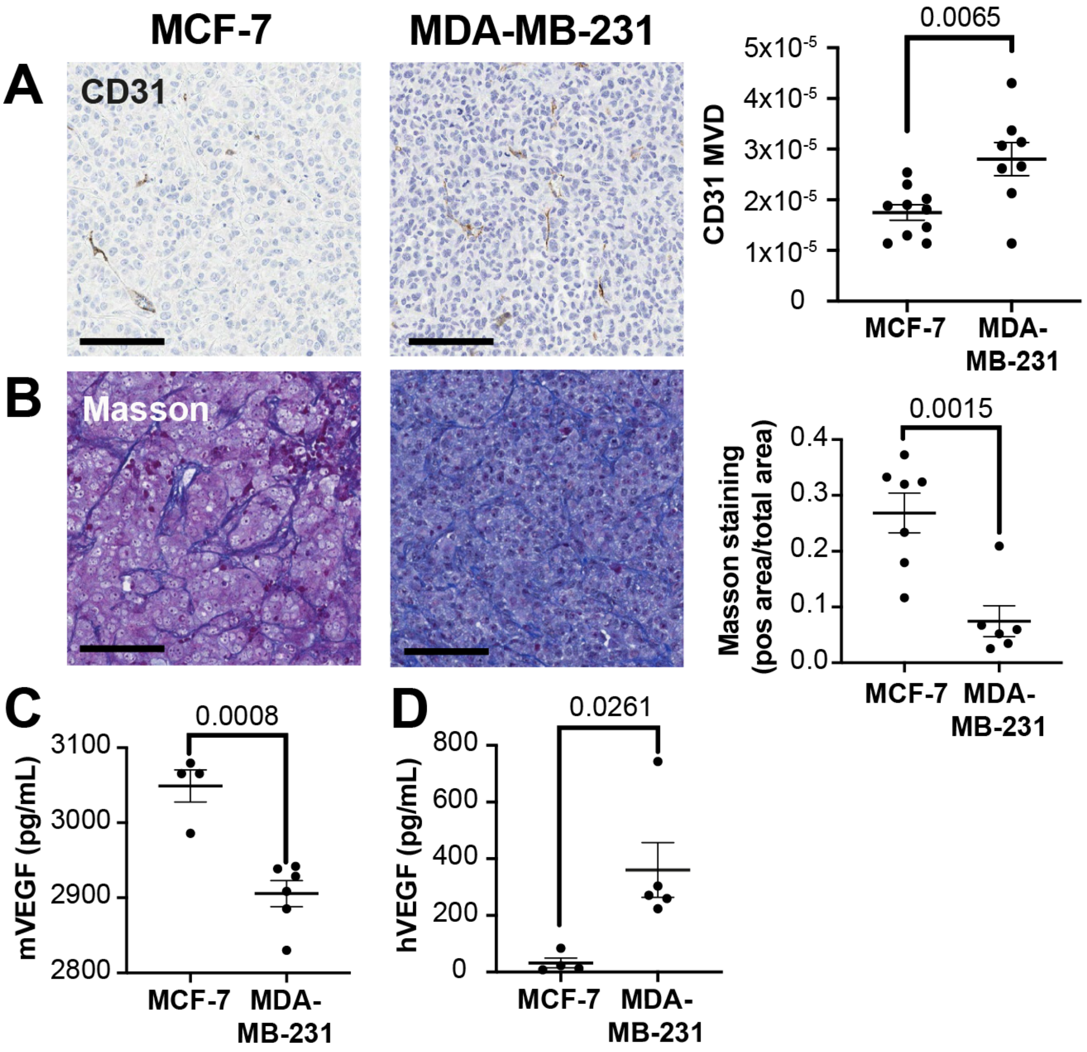

Supplementary Figure 3: Comparison of MCF-7 and MDA-MB-231 microenvironment at time of enrolment (tumour volume reaches 0.5 cm^3). (A) CD31 positive microvessel density (MVD, vessel/μm^2) was significantly higher for the MDA-MB-231 cohort compared to the MCF-7 cohort (MCF-7 $n_{tumours}$=10, MDA-MB-231 $n_{tumours}$=8); scale bar 100 μm. (B) Extracellular matrix stained by Masson's trichrome staining showing MDA-MB-231 tumours had significantly lower levels of collagen deposition than MCF-7 tumours (MCF-7 $n_{tumours}$=10, MDA-MB-231 $n_{tumours}$=8); scale bar 100 μm. (C) Circulating mouse VEGF (host source) is significantly higher in MCF-7 (MCF-7 n_{mice}=4, MDA-MB-231 n_{mice}=6). (D) Circulating human VEGF (tumour source) is significantly higher in MDA-MB-231 (MCF-7 n_{mice}=4, MDA-MB-231 n_{mice}=5). All panels, p-values displayed from 2-sided Student's t-tests except for (D) where a 2-sided Welch's t-test is performed due to unequal variances between the groups.

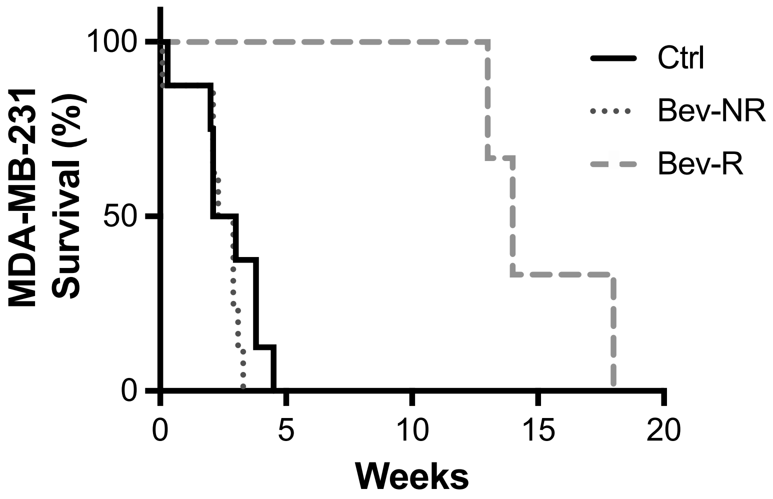

Supplementary Figure 4: Survival analysis for MDA-MB-231 tumours according to the non-responding and responding groups.

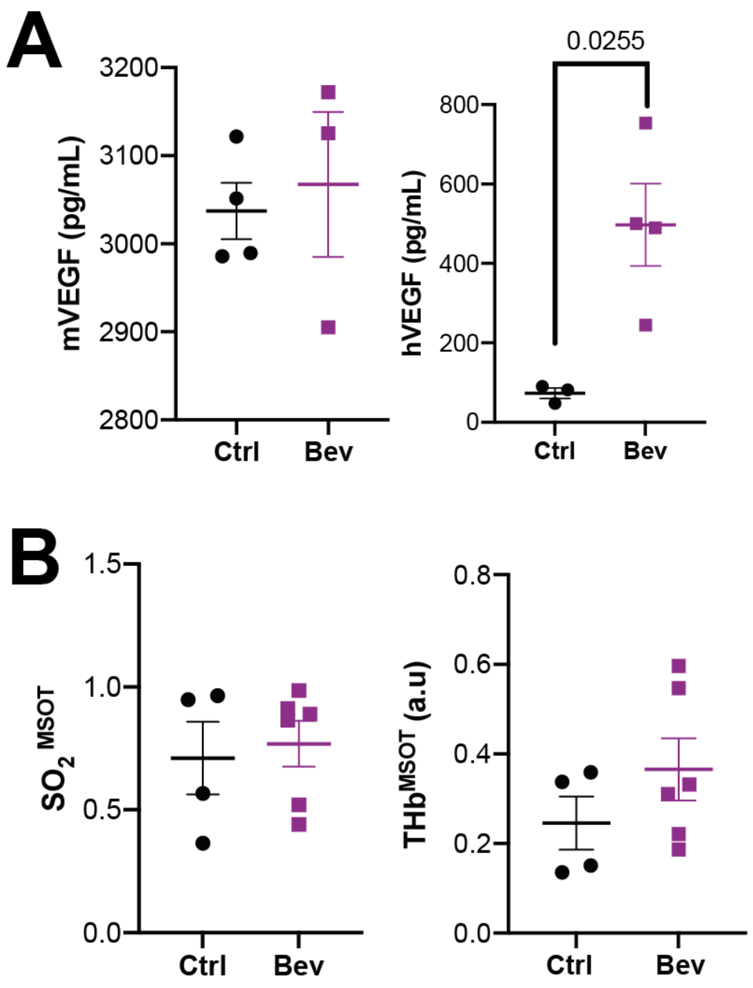

Supplementary Figure 5: MCF-7 Bevacizumab treatment response. (A) Circulating mouse VEGF (microenvironment source) shows no changes in response to Bev treatment (Ctrl n_{mice}=4, Bev n_{mice}=3), while circulating human VEGF (tumour source) is increases substantially after Bev treatment (Ctrl n_{mice}=3, Bev n_{mice}=4). (B) Tumour oxygenation (SO_2^{MSOT}) and haemoglobin content (THb^{MSOT}) extracted from PAT images of MCF-7 tumours shows no change in response to Bev treatment (Ctrl $n_{tumours}$=4, Bev $n_{tumours}$=6). All panels, p-values are displayed from a 2-sided Welch's t-test due to unequal variances.

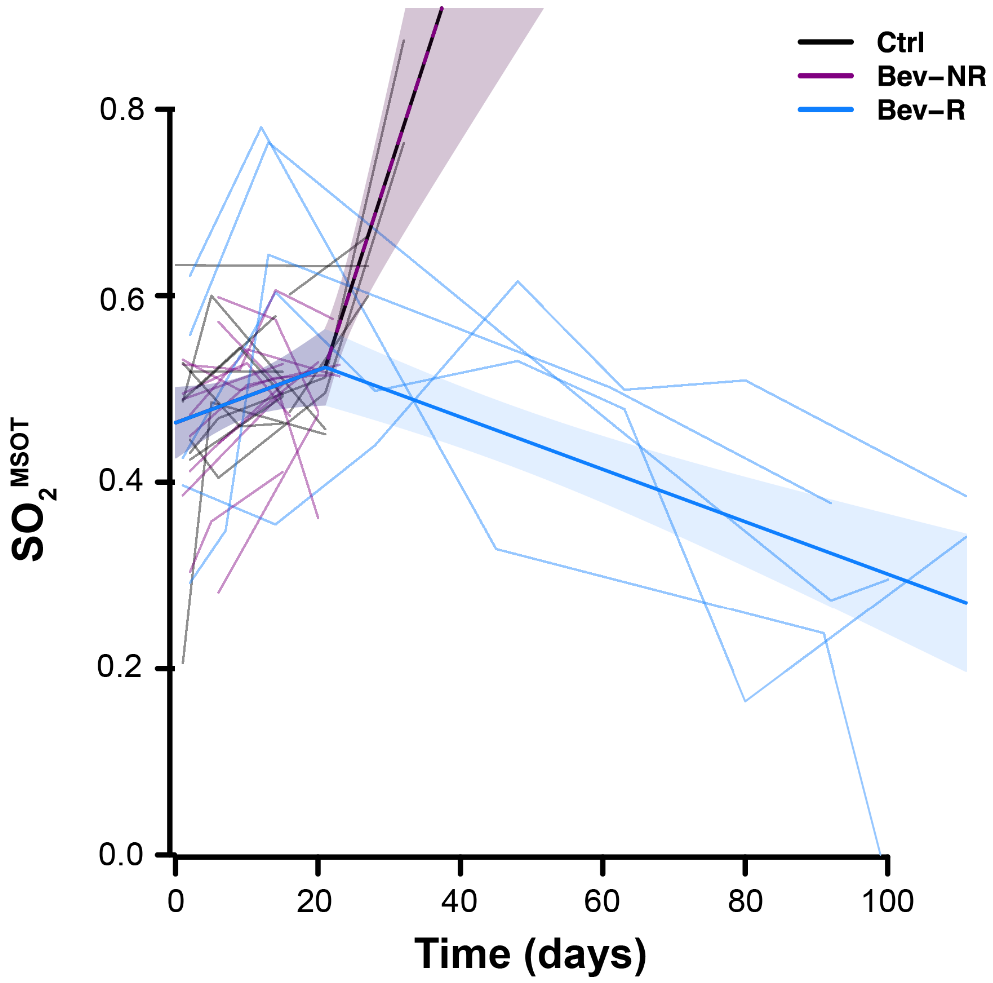

Supplementary Figure 6: Longitudinal analysis showing trajectories for each individual tumour. Duplicate of Figure 4D showing individual trajectories that contribute to the trendline.

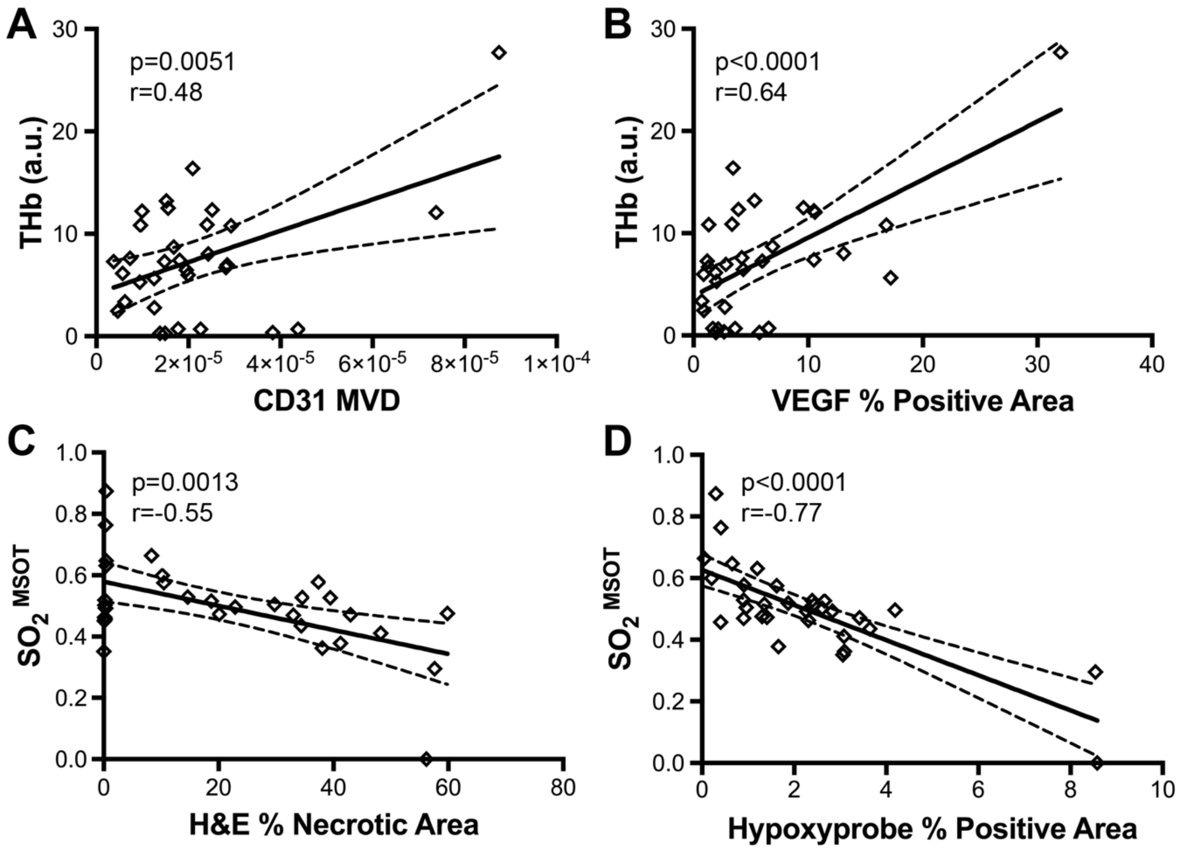

Supplementary Figure 7: Correlation analyses between *in vivo* PAT data and *ex vivo* immunohistochemistry data across all groups. (A) Correlation between PAT THb and CD31 microvessel density (MVD). (B) Correlation between THb and VEGF positive area. (C) Correlation between PAT SO_2^{MSOT} and necrotic area. (D) Correlation between SO_2^{MSOT} and hypoxyprobe positive area.

Supplementary Table 1. MDA-MB-231 mouse cohort (n=31). Mice marked in white and grey were included in the indicated experimental group. Mice marked in yellow, green and red were excluded. CON = control; BEV = Bevacizumab treated; Initial = tumours excised after imaging at the point of enrolment to provide a pre-treatment reference; Final = tumours monitored throughout treatment and excised at endpoint. Colour code: Grey - one tumour was absent (no growth), Red - both tumours were absent (no growth), Yellow - Mouse lost during PAT procedure, Green - Samples for histopathology or images from PAT could not be processed for further analysis.

Mouse ID	Group
BD21	BEV Final Left tumour only
BD22	BEV Final group
BD23	BEV Final group
BD24	CON Final group
BD25	BEV Final group
BD26	BEV Final group
BD27	CON Initial (pre-treatment) Group
BD28	BEV Final group
BD29	CON Final group
BD30	CON Final Left tumour only
BD31	Lost during procedure
BD32	CON Final group
BD33	CON Initial (pre-treatment) Group
BD34	BEV Final Left tumour only
BD35	CON Initial (pre-treatment) Group
BD36	CON Initial (pre-treatment) Group
BD38	CON Initial (pre-treatment) Group
BD39	BEV Final group
BD41	CON Final group
BD42	Lost during procedure
BD43	Faulty Histological or PAT
BD44	BEV Final group
BD45	CON Final group
BD46	BEV Final group
BD47	CON Final Left tumour only
BD49	CON Initial (pre-treatment) Group
BD50	No tumour growth
BC52	Faulty Histological or PAT
BC53	BEV Final Left tumour only
BC54	Faulty Histological or PAT
BC55	CON Final group
	**Total number of mice final group= 19**
	**Total number of tumours final group= 33**

Supplementary Table 2. MCF-7 mouse cohort (n=24). Mice marked in white and grey were included in the indicated experimental group. Mice marked in yellow, green and red were excluded. CON = control; BEV = Bevacizumab treated; Initial = tumours excised after imaging at the point of enrolment to provide a pre-treatment reference; Final = tumours monitored throughout treatment and excised at endpoint. Colour code: Red - Both tumours were absent (no growth), Orange - Estrogen pellet expired (i.e. beyond 90 days from implantation), Yellow - Mouse lost during PAT procedure or due to estrogen side effects, Green - Samples for histopathology or images from PAT could not be processed for further analysis.

Mouse ID	Group
BC21	BEV Final group
BC22	CON Initial (pre-treatment) group
BC24	Estrogen Loss
BC25	CON Final group
BC26	CON Final group
BC27	No tumour growth
BC28	BEV Final group
BC29	Estrogen Loss
BC30	Lost during experiment
BC31	Lost during experiment
BC32	CON Initial (pre-treatment) group
BC33	Faulty Histology or PAT
BC34	Lost during experiment
BC35	CON Initial (pre-treatment) group
BC36	CON Final group
BC37	CON Initial (pre-treatment) group
BC38	Faulty Histology or PAT
BC39	CON Initial (pre-treatment) group
BC40	Estrogen Loss
BC41	Estrogen Loss
BC42	Lost during experiment
BC43	CON Initial (pre-treatment) group
BC44	Estrogen Loss
BC45	Estrogen Loss
	**Total number of mice final group= 7**
	**Total number of tumours final group= 14**

Supplementary Table 3. Experimental numbers for the number of tumour samples contributing to each figure and panel in the main manuscript.

Figure	Panel	Description	Ctrl	Bev	Bev-NR	Bev-R
**1**	B	Survival curve MCF-7	4	4	-	-
	B	Survival curve MDA-MB-231	8	11	-	-
	C	Tumour growth MDA-MB-231	14	20	-	-
**2**	A & B	VEGF Elisa	5	-	6	3
	C	Biochemical Hb	7	-	8	2
	D	VEGF IHC	14	-	14	5
**3**	-	MSOT final	14	-	14	6
**4**	-	MSOT predictive	14	-	14	6
**5**	A	H&E Necrosis	14	-	13	4
	B	MVD (CD31 IHC)	14	-	13	5
	C	ASMA (IHC)	14	-	14	4
	D & E	Hypoxyprobe	13	-	14	5
	F	CD31/ASMA	14	-	14	4
	G	Tol Blue	14	-	14	5
	H	F4/80 (IHC)	14	-	14	5
